# An investigation into the optimal wear time criteria necessary to reliably estimate physical activity and sedentary behaviour from ActiGraph wGT3X+ accelerometer data in older care home residents

**DOI:** 10.1186/s12877-021-02725-6

**Published:** 2022-02-17

**Authors:** Jennifer Airlie, Anne Forster, Karen M. Birch

**Affiliations:** 1 Academic Unit for Ageing and Stroke Research, Bradford Institute for Health Research, Bradford Teaching Hospitals NHS Foundation Trust, Bradford, UK; 2 Academic Unit for Ageing and Stroke Research, Leeds Institute of Health Sciences, University of Leeds, Leeds, UK; 3 School of Biomedical Sciences, Faculty of Biological Sciences, University of Leeds, Leeds, UK

**Keywords:** Physical activity, Sedentary behaviour, Older adults, Care home residents, Accelerometer, ActiGraph wGT3X +, Measurement, Methodology, Reliability

## Abstract

**Background:**

Research protocols regarding the use of ActiGraph wGT3X+ accelerometers in care home residents are yet to be established. The purpose of this study was to identify the minimal wear time criteria required to achieve reliable estimates of physical activity (PA) and sedentary behaviour (SB) in older care home residents.

**Methods:**

Ninety-four older adults from 14 care homes wore an ActiGraph wGT3X+ accelerometer on the right hip for 7 consecutive days. A pragmatic, staged approach was adopted in order to explore the effect of: monitoring day; minimum daily wear time and number of wear days on estimates of four outcomes derived from the accelerometer data: counts^.^day^− 1^, counts^.^minute^− 1^, PA time and SB time.

**Results:**

Data from 91 participants (mean age: 84 ± 9 years, 34% male) was included in the analysis. No effect of monitoring day was observed. Lowering the daily wear time to ≥ 8 h (compared to ≥10 h) had no effect on the outcomes of interest. Four days of monitoring was sufficient to provide reliable estimates of all four outcomes.

**Conclusion:**

In this study, a minimum wear time criterion of ≥ 8 h on any 4 days was required to derive reliable estimates of PA and SB from ActiGraph wGT3X+ accelerometer data in older care home residents.

## Background

Over the previous century there has been a shift in the demographics of the world’s population [1], with a particular expansion of the 85 years and above age group (i.e. the oldest old). As longevity in later life improves [2] this trend is likely to continue and in the United Kingdom (UK), the number of individuals aged over 85 years is expected to more than double between 2014 and 2034 to 3.2 million [3].

Whilst population ageing may be viewed positively; the fact that increases in life expectancy are typically mirrored by extended periods of morbidity and disability cannot be overlooked [4–6]. Many older adults will experience complex and interacting health needs and will ultimately require some form of support in their later years [1]. Recent estimates suggest there are over 400,000 older adults residing in care homes in the UK [7]. In the UK care homes provide overnight accommodation and communal living facilities for long-term care for individuals who may not be able to live independently. Care homes also provide personal care, and in some instances nursing care, for those people with illness, disability or dependence [8].

By definition residents of care homes are amongst the frailest individuals in the UK population, distinguishable from community-dwelling older adults of the same age because of their possible physical limitations, multi-morbidity, dependency on others and / or cognitive impairment [9]. Still, frailty may be considered a dynamic process and whilst it is likely that care home residents become frailer and are at higher risk of worsening disability, falls and admission to hospital, this deterioration is not immutable and there is scope to intervene [10].

Indeed, there is now ample evidence that engagement in physical activity (PA) has beneficial effects on a range of outcomes related to health in older adults, including those residing in care homes [8, 11–13]. In addition, there is mounting evidence regarding the negative impact sedentary behaviour (SB), independent of PA, may have on a number of health parameters [14–16]. Nevertheless, research suggests older adults residing in care homes spend the majority of their time sedentary [17–19]. This suggests a shift in the emphasis of interventions from simply targeting PA to whole-home initiatives which look to encourage residents to engage in more routine PA and reduce the amount of time they spend being sedentary for prolonged periods of time would be well placed. However, in order to develop such interventions a thorough understanding of the levels and patterns of PA and SB in this population is needed.

Recent advances in PA and SB monitoring afford researchers the opportunity to further increase and refine understanding of these behaviours. Accelerometers in particular are increasingly being used in studies involving care home residents due to their capability to provide information not only on the total volume of PA (which is typically low in this population) and SB; but also, on the intensity of PA and the patterns of both PA and SB [20, 21].

Still, it is important to acknowledge that the complexity of accelerometers means the use of these devices is not without challenges [22]. Several decisions pertaining to the data collection and processing methods need to be made [23]. Whilst best practice recommendations have been published regarding accelerometer use [24, 25], the paucity of high-quality measurement specific research involving older adults means much of the evidence informing these recommendations is derived from studies with younger adults. Thus, whilst these offer a valuable resource, some of these recommendations may not be applicable to older adults residing in care homes.

Participants in habitual PA studies are typically asked to wear an accelerometer during all waking hours over a 7-day period [24, 25]; yet compliance to this protocol, particularly in older adults, is variable. We recently reported that 19% of older care home residents approached to wear an accelerometer declined and cited not wishing to wear the accelerometer for so many days as their reason for doing so [18]. Furthermore, reports of individuals forgetting to put the accelerometer back on after removing it for reasons such as showering (many of the monitors are not water-proof); discomfort and sleeping are not uncommon [26, 27]. Accordingly, researchers typically apply a minimum wear time (i.e. the proportion of a day and the number of days) which is required to ensure estimates of PA and SB are representative of habitual PA behaviour.

In studies involving community dwelling older adults a threshold of ≥ 10 h of accelerometer wear is widely used to define a valid day [28, 29]. Nevertheless, this threshold is not universally accepted [30] and it has been acknowledged that what constitutes a ‘day’ is likely to vary considerably between individuals. This point is particularly pertinent when considering older adults as they are such a heterogeneous group, with those residing in a care home tending to be frailer than their counterparts living in a community setting [9]. At the same time, it is probable that the variability in PA and SB across days is likely reduced in care home residents given there is often more structure to their daily routines [31]. This is supported by recent studies involving older adults in both a retirement community (mean age: 83.5 y ± 6.5 y) [32] and a care home setting (mean age = 82.6 y ± 9.2 y) [18] which report no difference in PA across days of the week. Consequently, whilst 5 days wear has previously been deemed necessary to ensure reliable estimates of PA behaviour in samples of community-dwelling older adults [33, 34], it may be the number of days of wear could be reduced in care home residents without distorting data.

Further work is required to investigate the minimum wear criteria necessary to ensure estimates of PA and SB in older adults living in care homes are reliable. Such work is warranted as the application of a minimum wear time criterion derived from studies involving different populations may result in either insufficient data being collected or data being needlessly excluded from analysis which can ultimately effect conclusions made.

Thus, the aim of this study was to identify the minimal wear time criteria required to achieve reliable estimates of PA and SB in older care home residents.

## Methods

### Participants

This study is based on data collected from older care home residents recruited from ten care homes enrolled in the Research Exploring Physical Activity in Care Homes (REACH) programme between June 2013 and March 2015 and four care homes involved in the associated development work between September 2011 and January 2012. All of the care homes were located in West Yorkshire. Ethical approval was obtained from the Yorkshire and The Humber - Bradford and East of England-Essex NHS research ethics committees (REACH programme) and the University of Leeds research ethics committee (development work).

All residents within each of the care homes were screened for eligibility. The eligibility criteria were as follows: aged 65 years or over; a permanent resident; not bed bound or in receipt of palliative care. A permanent resident was defined as someone who was residing in the care home and not in receipt of respite, day-care, or short-term rehabilitation.

An initial assessment of the mental capacity of all eligible residents was undertaken by the care home manager or an (appropriate) nominated person. As per the Mental Capacity Act (MCA) guidance, all residents were assumed to have capacity unless it was established that they did not [35]. The following question was used to guide this process: “Does the individual have the capacity to consent (or refuse) to take part in the research study at this point in time?” [36]. Highlighting that an individual’s mental capacity is “decision-specific” was deemed particularly important as it is possible that whilst an individual may be deemed unable to make a decision about their finances (as an example) they may be capable of making a decision about taking part in a research study. Prospective participants were only deemed to lack capacity if there was evidence that:

- they did not have a general understanding of the research project and what was expected of them (following the provision of information in an appropriate way, e.g. large print information sheets, verbal explanation of the research project);
- they were unable to retain the information long enough to be able to consider it and make an informed decision;
- they were unable to consider the potential benefits or risks of taking part in the research project;
- they were unable to communicate their decision [37].

In cases where the manager / nominated individual did not feel able to make a judgement on a resident’s capacity the researcher conducted this assessment.

Those deemed to have capacity were approached and, if they wished to participate, written or verbal (witnessed) informed consent was gained. For those residents who were considered not to have capacity, assent from a personal consultee (identified by the care home manager / nominated individual) or an Advanced Directive relevant to research was sought. If neither could be identified; the personal consultee did not feel able to take on the role or did not respond within the pre-determined timeframes, an appropriate member of staff (again identified by the care home manager / nominated individual) was asked to act as a nominated consultee.

### Procedures

An appropriate staff member within the care home (identified as someone who was familiar with the participants capabilities) was asked to complete the Barthel Index (BI) [38, 39]; the Functional Ambulation Classification (FAC) [40]; and report on the participants medical history (based on the Charlson Comorbidities Index (CCI) [41]. The following demographic information was also collected: gender, age, length of residence in the care home, height and weight.

Participants were asked to wear an ActiGraph wGT3X+ accelerometer (Actigraph, Pensacola, FL, USA) on the right hip, secured using an elasticated belt, during waking hours over the course of 7 days. Accelerometers were initialised to record raw data at a sampling rate of 30 Hz. No stop time was entered to allow for flexibility in wear time. Participants were advised that they were to remove the accelerometer if they were going to be engaging in any water-based activities (e.g. bathing) and were reassured that if they wished to remove the accelerometer for any reason (e.g. discomfort) they were permitted to do so. However, participants were encouraged to keep the accelerometer on during all waking hours for the duration of the monitoring period. Participants were also reassured that they did not need to “do anything” with the accelerometer other than wear it. A member of the research team visited the participant or spoke with a member of staff at the care home within one - two days of the accelerometer being fitted to ensure its proper use. Further contact with the participants and / or care home staff was undertaken only if needed.

For all participants, it was requested that a daily log of wear time (i.e. the time the monitor was put on and removed) was kept for the duration of the monitoring period. Participants capable of completing the log and putting the accelerometer on themselves were encouraged to do so; though the process of completing the activity log was also explained to staff and they were asked to offer support with this where appropriate.

### Accelerometery data processing

Raw acceleration data were downloaded and initially processed using the normal filter option and aggregated over 60 s epochs using the proprietary ActiLife software (version 6.8.0, ActiGraph Pensacola, Florida, USA). Vector magnitude (VM) counts were used for analysis. In the absence of consensus on the best approach to correctly identify and remove non-wear time from an accelerometer data set and in an effort to reduce the risk of distorting data provided by the least active participants [42], all data were manually screened (guided by the original rules detailed in Table 1) alongside the activity logs and periods of non-wear time were removed.

**Table 1:** Rules utilised to guide the manual screening of the accelerometer data to identified non-wear time

Accelerometer administration was indicated if one of the following conditions were met:	The removal of an accelerometer was indicated if one of the following conditions were met:
a) If 60 min of consecutive 0’s (with the allowance of a 5-min interruption in the string of consecutive zero’s) in the VM axis precedes a VA count value of ≥760 cpm (i.e. light intensity PA) then this value is assumed to indicate the accelerometer being put on. Note, if another light count is identified in the following 5 min use the latter count as the on time.	a) If the 60 min following a VA count value of ≥760 cpm (i.e. light intensity PA) contains is a string of consecutive 0’s (with the allowance of a 5-min interruption in the string of consecutive zero’s) in the VM axis.
b) If 120 min of consecutive 0’s (with the allowance of a 5-min interruption in the string of consecutive zero’s) in the VM axis precedes a VA count value of ≥100 cpm (i.e. low intensity PA) then this value is assumed to indicate the accelerometer being put on. Note, if another count ≥100 cpm identified in the following 5 min use the latter count as the on time	b) If the 120 min following a VA count value of ≥100 cpm (i.e. low intensity PA) contains is a string of consecutive 0’s (with the allowance of a 5-min interruption in the string of consecutive zero’s) in the VM axis.

*Abbreviations: VM *vector magnitude; *VA *vertical axis; *cpm *counts per minute

Daily wear time was determined by subtracting non-wear time from the total possible minutes in a day (1440 min). Data were then reviewed and the first monitoring day was removed if the monitor was administered after 1 pm. Partial days, defined as being < 4 h, were also removed as this amount of wear time was deemed insufficient to provide a reliable estimate of PA or SB outcomes.

In the current study the specific outcomes of interest were: total volume of PA (i.e. counts^.^day^− 1^ and counts^.^minute^− 1^), daily PA time and daily SB time. In the absence of cut-points developed specifically with care home residents, a published cut-point, developed in a sample of community-dwelling older adults (*n* = 37, mean age: 73.5 ± 7.3 y), was applied to the VM activity cpm to identify time spent engaging in PA (≥ 200 cpm) and SB (< 200 cpm) [43].

### Statistical analysis

All analyses were completed using SPSS version 21 (IBM Corporation, Somers, NY, USA). Data were first assessed for normality of distribution visually (via histograms) and using the Shapiro-Wilk test. In cases where data were non-normally distributed (i.e. counts^.^day^− 1^, counts^.^minute^− 1^ and daily PA time), data were log (log10) transformed prior to statistical testing to satisfy normality assumptions and permit the use of parametric statistics. Data are presented as mean ± standard deviation (SD) unless otherwise specified and significance was defined as *p* < 0.05.

In order to explore the impact of different wear time criteria on the assessment of key accelerometer outcomes (i.e.counts^.^day^− 1^, counts^.^minute^− 1^, PA time and SB time) a pragmatic, staged approach was adopted as follows:

#### The effect of monitoring day on accelerometer outcomes

Given the hierarchical structure of the data (i.e. repeated measures within participants) and the fact that not all participants had an equal number of repeated measures (i.e. not all had data on 7 days), linear mixed effect models were used to explore the effect of monitoring day on the accelerometer outcomes. As wear time and sedentary time are related, all data were adjusted for daily wear time [44]. Each model included a random intercept for participants and care home to account for the nesting of observations within participants and the clustering of participants within care homes.

#### The impact of differing minimum daily wear time criteria on accelerometer outcomes

Wear times of 6 h, 7 h, 8 h, 9 h and 10 h were compared. Only data from participants who provided ≥ 1 day of data with a minimum daily wear time of ≥ 10 hours over the course of the measurement period were included in this section of analysis to ensure the sample was consistent. Linear mixed effect models were conducted for each accelerometer outcome as not all participants had seven days of data. The models included a random intercept for participants and care home to account for the nesting of observations within participants and the clustering of participants within care homes. Where the outcomes differed between the minimum daily wear time criteria, Bonferroni corrected pairwise comparisons were conducted. Recognising that it is not appropriate to conclude that the outcomes of interest are the same / equivalent based solely on the absence of a statistical difference, tests of equivalence using a confidence approach were also untaken to demonstrate the comparability between estimates of the outcomes of interest when different minimum daily wear time criteria were used [45]. The threshold of equivalency was set at ±10% of the mean of each of the PA outcomes (i.e. counts^.^day^− 1^, counts^.^minute^− 1^, PA time and SB time) when minimum daily wear time was ≥10 h as this was chosen as the criterion. Given that accelerometry data presents as both direct and derived measures, a 10% threshold of PA time equated to ±14 min. We felt that for this population a difference of 14 min spent in PA over a day would be practically meaningful.

#### The impact of number of monitoring days on the reliability of accelerometer outcomes

Data from participants who provided 7 valid days were averaged over an increasing number of days of data collection (i.e. day one average, day one and two average, day one, two and three average etc.). Linear mixed effect models were then conducted to explore the effect of varying the number of monitoring days. The models included a random intercept for participants and care home to account for the nesting of observations within participants and the clustering of participants within care homes. Where the accelerometer outcomes differed according to the number of monitoring days, Bonferroni corrected pairwise comparisons were conducted. The magnitude of the difference in the accelerometer outcomes based on fewer days of monitoring was also determined using standardised effect size [46]. In addition, tests of equivalence using a confidence approach [45] were untaken to demonstrate the comparability between the estimates of the outcomes of interest based on a different number of monitoring days. The threshold of equivalency was set at ±10% of the mean of each of the PA outcomes (i.e. counts^.^day^− 1^, counts^.^minute^− 1^, PA time and SB time) based on 7 days of monitoring as this was chosen as the criterion.

## Results

### Sample characteristics

A hip-worn accelerometer was administered to 94 participants. However, data from three participants were not considered further as it did not meet the initial screening criteria (i.e. 1 day with a minimum daily wear time of ≥ 4 hours). Thus, the analysis sample comprised 91 participants. Personal characteristics did not differ between those participants not included in the analysis sample and those who met the initial screening criteria (*p* > 0.05). The personal characteristics of those participants included in the analysis (*n* = 91) are presented in Table 2.

**Table 2:** Participant characteristics (n = 91)

	***n***	N (%) or Mean ± SD
Gender (male)	91	31 (34%)
Age (y)	85	84 ± 9
Age group	85	
< 85 y		39 (46%)
≥ 85 y		46 (54%)
Length of residence (months)	85	35 ± 65
Height (cm)	72	161.9 ± 10.7
Weight (kg)	83	66.7 ± 15.4
Capacity to consent (yes)	91	68 (75%)
Number of comorbidities^a^:	73	
None		4 (5%)
1–2		54 (74%)
≥ 3		15 (21%)
BI Score (score on a 21-point scale; 0–20)	86	12 ± 5
BI score ≤ 11 (dependent)		38 (44%)
BI score > 11 (independent)		48 (56%)
FAC	80	
Level 0 (non-functional ambulation)		11 (14%)
Level 1 (ambulatory-dependent for physical assistance – level II)		9 (11%)
Level 2 (ambulatory-dependent for physical assistance – level I)		4 (5%)
Level 3 (ambulatory-dependent for supervision)		3 (4%)
Level 4 (ambulatory-independent on level surfaces)		34 (42%)
Level 5 (ambulatory-independent)		19 (24%)

Notes: Number of participants (*n*) is not equal to the total number of residents recruited due to missing data; ^a^ based on the Charlson Comorbidity Index

*Abbreviations: BI *Barthel Index; *FAC *Functional Ambulation Classification

### Effect of monitoring day on accelerometer outcomes

**Fig. 1:**
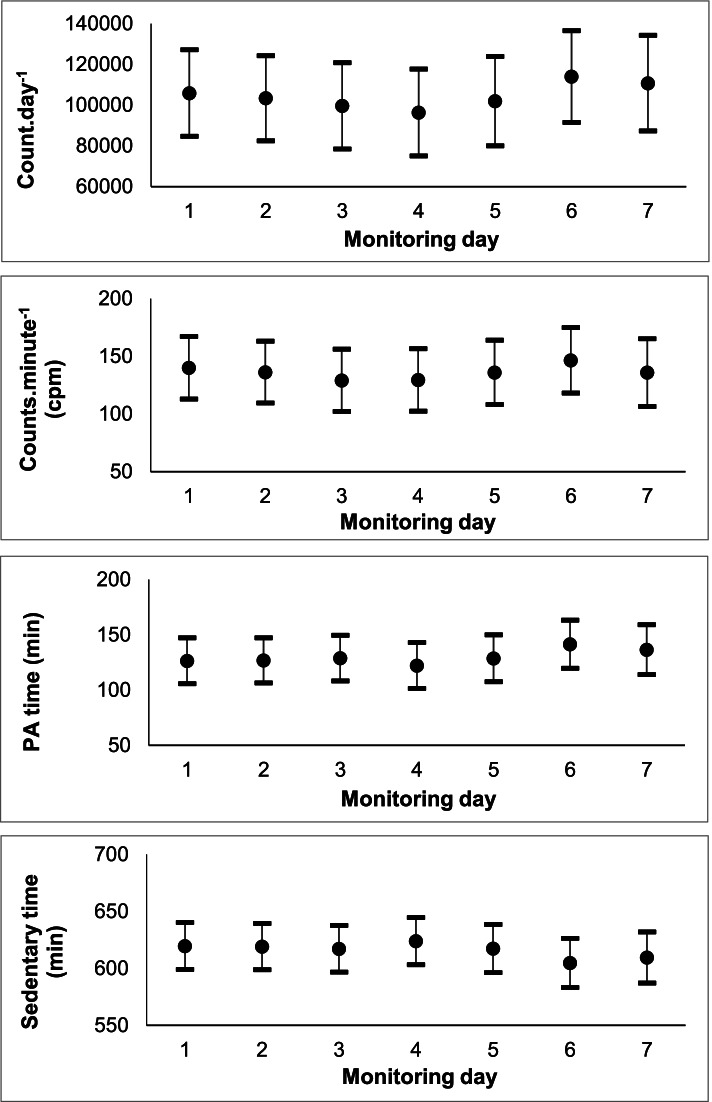
Mean and 95% confidence intervals of (reading top to bottom): counts.day^− 1^, counts.minute^− 1^, physical activity (PA) time and sedentary behaviour (SB) time across monitoring days (one-seven) (*n* = 91)

Estimates of the accelerometer outcomes (i.e. counts^.^day^− 1^, counts^.^minute^− 1^, PA time and SB time) across monitoring days are displayed in Fig. 1. Recorded accelerometer outcomes did not differ with monitoring day (*p* > 0.05).

### Impact of differing minimum daily wear time criteria on accelerometer outcomes

Data from participants (*n* = 85) who provided 1 day of data with a minimum daily wear time of ≥10 h were included in this section of analysis. The number of days available for analysis increased as the minimum number of hours used to define a valid day decreased. As can be seen in Table 3, population estimates of counts^.^day^− 1^, counts^.^minute^− 1^ and PA time were similar, irrespective of the minimum daily wear time criteria employed (*p* > 0.05). Conversely, estimates of SB time were significantly lower when valid days were defined as ≥7 h (Mean difference: − 21 min) or ≥ 6 h (Mean difference: − 26 min) compared to ≥ 10 h (*p* < 0.05) (Table 3).

**Table 3:** Accelerometer outcomes calculated based on different definitions of a valid day and equivalence results

Minimum daily wear time criteria (h)	Mean (95% CI)	Mean Difference	Standard Error	90% CI around mean difference	
				Lower	Upper
**counts.day**^**− 1**^					
≥ 10 h (EI: ± 11,170)	111,695 (90,870, 132,520)	n/a	n/a	n/a	n/a
≥ 9 h	109,297 (88,545, 130,049)	2398	3864	− 7563	12,359
≥ 8 h	107,976 (87,262, 128,690)	3719	3821	− 6132	13,569
≥ 7 h	107,569 (86,870, 128,269)	4126	3802	− 5676	1392
≥ 6 h	107,054 (86,364 127,744)	4641	3789	− 5127	14,409
**counts.minute**^**−1**^ **(cpm)**					
≥ 10 h (EI: ± 14)	140 (114, 166)	n/a	n/a	n/a	n/a
≥ 9 h	140 (113, 166)	0	4	−10	11^b^
≥ 8 h	139 (113, 165)	1	4	−10	11^b^
≥ 7 h	139 (113,166)	1	4	−10	11^b^
≥ 6 h	139 (113,165)	1	4	−10	11^b^
**PA time (h and min)**					
≥ 10 h (EI: ± 14 min)	2 h 20 min (1 h 58 min, 2 h 41 min)	n/a	n/a	n/a	n/a
≥ 9 h	2 h 17 min (1 h 55 min, 2 h 38 min)	3 min	3 min	- 6 min	12 min^b^
≥ 8 h	2 h 15 min (1 h 53 min, 2 h 36 min)	5 min	3 min	- 4 min	14 min^b^
≥ 7 h	2 h 14 min (1 h 52 min, 2 h 36 min)	6 min	3 min	- 3 min	14 min^b^
≥ 6 h	2 h 13 min (1 h 52 min, 2 h 35 min)	6 min	3 min	- 2 min	15 min
**SB time (h and min)**					
≥ 10 h (EI: ± 65 min)	10 h 54 min (10 h 25 min, 11 h 20 min)	n/a	n/a	n/a	n/a
≥ 9 h	10 h 41 min (10 h 14 min, 11 h 8 min)	12 min	7 min	- 6 min	30 min^b^
≥ 8 h	10 h 35 min (10 h 8 min, 11 h 2 min)	17 min	7 min	- 1 min	35 min^b^
≥ 7 h	10 h 31 min (10 h 4 min, 10 h 58 min)	21 min^a^	7 min	4 min	39 min^b^
≥ 6 h	10 h 29 min (9 h 59 min, 10 h 53 min)	26 min^a^	7 min	8 min	44 min^b^

Notes: Accelerometer outcomes are: counts.day^− 1^, counts.minute^− 1^, PA time and SB time. Mean difference from the 10 h reference point. ^a^ denotes that the mean difference from the estimates based on 10 h wear time criteria is significant at the 0.05 level. Bonferroni correction applied. Equivalence interval = ± 10% of mean value when the minimum daily WT of ≥10 h was used. For counts.day^− 1^ = 11,170 count.day^− 1^; counts.minute^− 1^ = 14 count^.^minute^− 1^; PA time = 14 min; SB time = 65 min. ^b^ indicates equivalency

*Abbreviations: EI *equivalency interval; *cpm *counts per minute; *PA *physical activity; *SB *sedentary behaviour

Table 3 also presents the equivalency test results for each of the daily minimum wear time criteria. These results show that estimates of counts^.^minute^− 1^ and SB time were equivalent to estimates based on the reference minimum wear time criteria of ≥ 10 h irrespective of the minimum daily wear time employed. For PA time, estimates were equivalent to those based on the reference minimum wear time criteria of ≥ 10 h for the ≥ 9 h, ≥ 8 h and ≥ 7 h criterion. Conversely, none of the estimates of counts^.^day^− 1^ based on alternative daily minimum wear time criteria were equivalent to those based on the reference minimum wear time criteria of ≥ 10 h. Setting the equivalency interval at ±15% of the mean of the counts^.^day^− 1^ (i.e. ± 16,754 counts^.^day^− 1^) revealed all estimates to be equivalent to the reference.

### The impact of number of monitoring days on the reliability of accelerometer outcomes

Data from participants (*n* = 35) who provided 7 days of data with a minimum daily wear time of ≥ 8 h were included in this section of analysis. Importantly these participants did not differ from those who did not meet this wear time criterion (Table 4, *p* > 0.05).

**Table 4:** Characteristics of participants stratified according to whether they met the minimum wear time criteria^a^

Did participants provide 7 days with a minimum daily wear time of ≥ 8 h?	Yes (n = 35)		No (***n*** = 59)	
	***n***	N (%) or Mean ± SD	***n***	N (%) or Mean ± SD
Gender (male)	35	12 (34%)	59	20 (34%)
Age (y)	31	83 ± 8	56	85 ± 9
Length of residence (months)	31	44 ± 97	56	30 ± 34
Height (cm)	28	161.3 ± 10.3	46	162.3 ± 10.9
Weight (kg)	30	67.8 ± 17.8	55	65.9 ± 13.9
Capacity to consent (yes)	35	24 (69%)	59	46 (78%)
Number of comorbidities^b^:	28		47	
None		1 (4%)		3 (6%)
1–2		21 (75%)		35 (75%)
≥ 3		6 (21%)		9 (19%)
BI score (score on a 21-point scale; 0–20)	35	13 ± 5	54	12 ± 6
BI score ≤ 11 (dependent)		15 (43%)		23 (43%)
BI score > 11 (independent)		20 (57%)		31 (57%)
FAC	31		51	
Level 0 (non-functional ambulation)		1 (3%)		10 (20%)
Level 1 (ambulatory-dependent for physical assistance – level II)		3 (10%)		6 (12%)
Level 2 (ambulatory-dependent for physical assistance – level I)		1 (3%)		3 (6%)
Level 3 (ambulatory-dependent for supervision)		1 (3%)		2 (4%)
Level 4 (ambulatory-independent on level surfaces)		16 (52%)		19 (37%)
Level 5 (ambulatory-independent)		9 (29%)		11 (21%)

Notes: ^a^ Wear time criteria defined as ≥ 8 h on 7 days. Number of participants (n) is not equal to the total number of residents recruited due to missing data. ^b^ based on the Charlson Comorbidity Index

*Abbreviations: BI *Barthel Index; *FAC *Functional Ambulation Classification

Estimates of the accelerometer outcomes derived by averaging over an increasing number of repeated days are presented in Table 5. Counts^.^minute^− 1^ was unaffected by the number of monitoring days included in analysis (*p* > 0.05). However, the number of monitoring days included in analysis did impact estimates of counts^.^day^− 1^ (F [6] = 2.713, *p* = 0.05); PA time (F [6] = 4.641, *p* < 0.01) and SB time (F [6] = 22.013, *p* < 0.01). For counts^.^day^− 1^ and PA time, only the estimate based on 1 monitoring day differed significantly from the 7-day average (*p* < 0.05). Estimates of SB time based on 1, 2 and 3 days of monitoring all significantly differed from the 7-day average (*p* < 0.05). Estimates based on a minimum of 4 days and above did not (*p* > 0.05).

**Table 5:** Accelerometer outcomes calculated based on an increasing number of days of data collection and equivalence results

**Counts.day**^−1^							
Day (n)	1	2	3	4	5	6	7 (EI: ± 14,151)
Mean ± SD	124,046 ± 85,406	140,581 ± 94,191	137,584 ± 97,130	136,500 ± 96,012	136,262 ± 93,744	139,427 ± 98,997	141,510 ± 104,549
Mean difference^b^	17463^a^	929	3926	5010	5248	2082	n/a
Effect Size	0.18	0.01	0.04	0.05	0.05	0.02	n/a
90% CI around mean difference							
Lower	3295	−13,239	−10,242	− 9158	− 8920	− 12,085	n/a
Upper	31,631	15,097	18,093	19,177	19,415	16,250	n/a
**Counts**^**.**^**minute**^**−1**^ **(cpm)**							
Day (n)	1	2	3	4	5	6	7 (EI: ± 18)
Mean ± SD	181 ± 123	186 ± 129	174 ± 123	171 ± 124	170 ± 119	172 ± 123	172 ± 125
Mean difference^b^	9	14	2	1	2	0	n/a
Effect Size	0.08	0.11	0.02	0.01	0.02	0.00	n/a
90% CI around mean difference							
Lower	- 24	- 28	−17	- 13	- 12	- 14	n/a
Upper	4^b^	1^b^	12^b^	15^b^	17^b^	14^b^	n/a
**PA time (h and min)**							
Day (n)	1	2	3	4	5	6	7 (EI: ± 18 min)
Mean ± SD	2 h 27 min ± 1 h 34 min	2 h 44 min ± 1 h 40 min	2 h 45 min ± 1 h 46 min	2 h 45 min ± 1 h 46 min	2 h 45 min ± 1 h 44 min	2 h 48 min ± 1 h 45 min	2 h 50 min ± 1 h 46 min
Mean difference^†^	23 min^a^	6 min	5 min	5 min	5 min	2 min	n/a
Effect Size	0.23	0.06	0.05	0.04	0.04	0.02	n/a
90% CI around mean difference							
Lower	9 min	- 8 min	- 9 min	- 8 min	- 9 min	- 12 min	n/a
Upper	37 min	20 min	19 min	19 min	19 min	16 min^b^	n/a
**SB time (h and min)**							
Day (n)	1	2	3	4	5	6	7 (EI: ± 1 h 4 min)
Mean ± SD	9 h 3 min ± 2 h 2 min	9 h 50 min ± 2 h 2 min	10 h 15 min ± 1 h 51 min	10 h 28 min ± 1 h 51 min	10 h 34 min ± 1 h 51 min	10 h 35 min ± 1 h 49 min	10 h 38 min ± 1 h 45 min
Mean difference^b^	1 h 35 min^a^	48 min^a^	23 min	10 min	5 min	3 min	n/a
Effect Size	0.83	0.44	0.21	0.09	0.04	0.03	n/a
90% CI around mean difference							
Lower	1 h 6 min	19 min	- 6 min	- 19 min	- 25 min	- 27 min	n/a
Upper	2 h 4 min	1 h 17 min	52 min^b^	39 min^b^	34 min^b^	32 min^b^	n/a

Notes. Accelerometer outcomes are: counts.day^− 1^, counts.minute^− 1^, PA time and SB time. A valid day being defined as a having a wear time of ≥ 8 h. Mean difference from the seven-day reference point. ^a^ denotes that the mean difference from the estimate based on 7 days is significant at the 0.10 level. Bonferroni correction applied. Equivalence interval = ± 10% of mean when the minimum daily WT of ≥10 h was used. For counts.day^− 1^ = 14,151 count.day^− 1^; cpm = 18 cpm; PA time = 18 min; SB time = 1 h 4 min. ^b^ indicates equivalency

*Abbreviations: CI *confidence interval; *PA *physical activity; *SB* sedentary behaviour; *cpm *counts per minute

Table 5 also presents the equivalency tests based on differing number of monitoring days. These results show that estimates of counts^.^minute^− 1^ were equivalent to estimates based on the reference of 7 monitoring days irrespective of the number of monitoring days included in the analysis. For SB time estimates based on 3, 4, 5  and 6 days of monitoring were equivalent to the criterion of 7 days. However, for PA time, only the estimate based on at least 6 days was equivalent to the estimate based on reference of 7 days. For counts^.^day^− 1^, none of the estimates based on fewer monitoring days were equivalent to those based on a minimum of 7 days of monitoring. When the equivalency interval was set at ±15% of the 7-day mean for both counts^.^day^− 1^ (i.e. ± 21,227 counts^.^day^− 1^) and PA time (i.e. ± 26 min), estimates based on 2, 3, 4, 5 and 6 days were equivalent to estimates based on the reference of 7 days of monitoring.

## Discussion

Although accelerometers are increasingly being used with care home residents [17, 18, 47], to our knowledge the current study is the first to explore the minimal wear time criteria necessitated to achieve reliable estimates of PA and SB in this population.

In the absence of a consensus on how many hours of wear constitutes a valid day it seemed prudent to explore whether population estimates of PA and SB varied dependent on the criteria used to define a valid day in care home residents. In order to do this the current paper adopted two methods of demonstrating comparability to the chosen criterion (i.e. ≥ 10 h): significance testing (which is often used within the literature) and equivalency testing (the more appropriate approach). We provided both methods for discussion. For count^.^minute^− 1^ the results from both methods indicated that estimates were equivalent irrespective of the minimum daily wear time employed. For SB time estimates were significantly lower when the minimal wear time criteria was lowered from ≥10 h (reference) to ≥ 7 h or ≥ 6 h (*p* < 0.05). However, equivalency tests demonstrated that estimates were equivalent irrespective of the minimum daily wear time employed. For counts^.^day^− 1^ and PA time estimates did not differ significantly when the minimal wear time criteria was lowered; however, estimates based on different minimal wear time criteria were not always equivalent to the reference (i.e. ≥ 10 h). For PA time the estimate based on a minimal wear time criteria of ≥ 6 h was not equivalent to the reference. Conversely, for counts^.^day^− 1^, none of the estimates based on lower wear time criteria were deemed to be equivalent to the reference at a threshold of 10%. Still, it is interesting to note that when the equivalency interval was set at ±15% estimates were equivalent, irrespective of the minimum daily wear time employed.

It is important to note that there are no set standards for setting an equivalency interval, rather it is suggested that an equivalency interval is selected based on practical relevance and strong rationale [48]. Given accurate measurement of both PA and SB outcomes is imperative, measurement error must be a consideration. Whilst the exact measurement error for the PA outcomes reported on is unknown, reports suggest it may be upwards of 20–30% at an individual level [49]. In light of this it is important to acknowledge that whilst setting the equivalency interval to ±10% may be deemed appropriate for outcomes such as PA time and SB time it may not be appropriate for counts.day^− 1^.

Based on the results of the current study an argument can be made for reducing the minimum wear time criteria used to define a valid day. Whilst lower thresholds, most notably ≥ 8 h of accelerometer wear, have been used previously in studies with older adults (including those residing in care homes) [17, 47, 50] the current study is the first to provide empirical evidence to support this decision.

While it may be surmised that a 7-day monitoring protocol may be too burdensome for older care home residents [18] there is a concern that using fewer days would lead to inaccurate estimates of PA and SB if there is considerable variation between days. However, it may be inferred that the variability in PA across days is likely reduced in care home residents given the functional impairments characteristic of this population and structured routine typical of a care home setting [9, 31]. The results presented in the current study support this hypothesis, as key outcomes (i.e. counts^.^day^− 1^, counts^.^minute^− 1^, PA time and SB time) were consistent across monitoring days. These findings are in accordance with recent studies conducted in both a retirement community and care home setting which reported no difference in outcomes across days of the week [18, 31]. Moreover, in the present study, estimates of accelerometer outcomes based on as few as 4 days of monitoring did not differ significantly from estimates based on 7 days (*p* > 0.05). Taken together these findings suggest it is not necessary to be prescriptive regarding the ‘type’ of day (i.e. weekend day or weekday) included in analysis and that the number of days wear could be reduced without distorting data.

Caution is warranted when considering whether to reduce the number of monitoring days. Based on the equivalency results the number of days of monitoring deemed necessary would differ dependent on the outcome of interest. For example, whilst estimates for SB time based on 3, 4, 5 and 6 days of monitoring were deemed equivalent to the estimate based on the reference of 7 days, for PA time, only the estimate based on at least 6 days was deemed equivalent. In light of this it is recommended that whenever possible the 7-day monitoring period should be implemented.

### Limitations

In considering the findings presented it is important to acknowledge the current study is not without limitations. Firstly, the characteristics and PA and SB profile of the participants included will have had an impact on the findings therefore the results are only likely to be relevant to “similar” populations. However, participants were resident in a number of care homes in different social-economic areas and demographic data suggest they are similar to residents of care homes reported elsewhere [16, 51, 52]. In addition, there were no differences in the participant characteristics between those participants who were included in the analysis sample and those who were not.

Secondly, four specific accelerometer outcomes (i.e. counts^.^day^− 1^, counts^.^minute^− 1^, PA time and SB time) were considered therefore the results presented are specific to these. It is not appropriate to assume that the minimal wear time criteria proposed would be sufficient to achieve reliable estimates of different outcomes. Previous research conducted in adults and older adults suggests the number of valid days needed to achieve reliable estimates of PA decreases as the intensity of the PA increases [34, 53, 54]. This may be attributable to the fact engagement in moderate-vigorous (MV) PA tends to be planned therefore is less variable. Although estimates of differing intensities of PA were not considered in the present study, it may be postulated that the reverse would be true for care home residents. Any engagement in higher intensity PA is likely to be sporadic thus more valid days would be required to ensure estimates were reliable. Nevertheless, given the profile of PA and SB in older care home residents [17, 18], the outcomes included in this study were deemed to be most relevant to this population. Furthermore, in the absence of a consensus on wear time criteria and lack of empirical evidence supporting the superiority of one criterion over another, the current study offers a considerable contribution to the existing literature. It is however important to recognise that the normal filter was used when processing the accelerometer count data which may resulted in an underestimation of PA time and overestimation of SB time given the cut point applied was derived using the low-frequency extension filter.

Finally, it is important to recognise that, as a consequence of the pragmatic, staged approach adopted, inferences made about the minimum accelerometer wear time criteria required to achieve reliable estimates of key accelerometer outcomes of interest in this population were made based on analysis conducted with smaller sub-groups of the larger analysis sample which could lead to concerns around bias. For example, when exploring whether the number of days of monitoring could be reduced, only participants who had 7 complete days of data were included in the analysis. In light of this, whilst there were no differences in the measured characteristics of participants included and those who were not in any of stage of the analysis, future studies may want to consider undertaking some statistical simulation work.

### Implications

In the absence of best practice recommendations regarding the use of accelerometers in field-based research with older care home residents the empirical evidence provided in the current study can help researchers design accelerometer data collection protocols and facilitate decision making. For example, reducing participant burden is a particularly pertinent consideration in studies with older care home residents given the fragility of the population therefore a researcher may wish to use a shorter monitoring period. However, the results of the current study support the continued use of a 7-day monitoring period wherever possible in an effort to ensure sufficient data is collected to be confident that estimates of PA and SB are accurate. Having said this, undertaking research in a care home setting is particularly challenging therefore it is important to acknowledge that there may be cases where participants are not fully compliant with the data collection protocol. Hence, the application of a population specific minimum wear time criterion is beneficial as it maximises the sample size and subsequently the volume of useful accelerometer data retained in cases where a substantial proportion of participants do not present 7 days’ worth of data.

## Conclusion

Determining the volume of data required to reliably estimate PA and SB whilst minimising participant burden and ensuring compliance is challenging. A balance between measurement reliability and sample size is needed to warrant confidence in the outcomes reported. In adopting a pragmatic approach, this study demonstrated that the minimum daily accelerometer wear time could be reduced in care home population without affecting the reliability of estimates of key PA and SB outcomes (i.e. counts^.^day^− 1^, counts^.^minute^− 1^, PA time and SB time) in a care home population. The results also suggest the number of days of wear necessary to ensure estimates are reliable could be reduced however this may be dependent on the outcome of interest. Accordingly a 7-day monitoring protocol should be utilised wherever possible to increase the likelihood of participants meeting the minimal wear time requirements and increase the chances of achieving reliable estimates of various outcomes simultaneously.

## Data Availability

The dataset generated and analysed during the current study are available from the corresponding author on reasonable request.

## Abbreviations

BI: Barthel Index
CCI: Charlson Comorbidity Index
CPM: counts per minute
FAC: Functional Ambulation Category
Hz: Hertz
MCA: Mental Capacity Act
MVPA: moderate-vigorous physical activity
NIHR: National Institute for Health Research
PA: physical activity
PAG: physical activity guidelines
REACH: Research Exploring physical Activity in Care Homes
SB: sedentary behaviour
SD: standard deviation
UK: United Kingdom
VA: vertical axis
VM: vector magnitude
WHO: World Health Organisation
